# Coral connectivity between equatorial eastern Pacific marine protected areas: A biophysical modeling approach

**DOI:** 10.1371/journal.pone.0202995

**Published:** 2018-08-29

**Authors:** Bertrand D. Lequeux, Miguel-Angel Ahumada-Sempoal, Andrés López-Pérez, Cristóbal Reyes-Hernández

**Affiliations:** 1 Program in Marine Biology, Universidad del Mar, Ciudad Universitaria s/n, Puerto Ángel, San Pedro Pochutla, Oaxaca, México; 2 Universidad del Mar, Ciudad Universitaria s/n, Puerto Ángel, San Pedro Pochutla, Oaxaca, México; 3 Universidad Autónoma Metropolitana, Unidad Iztapalapa, colonia Vicentina, Ciudad de México, México; Department of Agriculture and Water Resources, AUSTRALIA

## Abstract

There are many marine protected areas (MPAs) containing coral reef aggregations in the eastern Pacific region. However, the connectivity of corals between MPAs is still poorly known, especially in the Marine Conservation Corridor of the Eastern Tropical Pacific (MCCETP). Here, we assess the potential connectivity of corals across equatorial eastern Pacific MPAs through a Lagrangian particle-tracking algorithm coupled offline with an ocean-circulation numerical model. Connectivity metrics and graph theory were used to analyze the networks and highlight those MPAs that are critical for maintaining the connectivity of corals across the region. Our results show that the equatorial eastern Pacific MPAs form a relatively well-connected network, at least 40% of coral larvae released per year end up within the boundaries of an MPA. MPAs like Malpelo and Gorgona islands included in the MCCETP were found to be critical for connectivity of corals because of their high betweenness centrality and potential role as stepping-stones between coastal MPAs and offshore MPAs such as the Galapagos Islands. Two pelagic larval duration (PLD) scenarios (40 and 130 days) indicate a quasi-unidirectional larval flow from coastal MPAs toward oceanic MPAs, where the only resilient MPAs (Coiba and Malpelo islands) depend mostly on subsidiary recruitment from MPAs located along the coast of Costa Rica, Panama and Colombia. In the two PLD scenarios, Cocos Island maintains a very low resilience potential. Our results indicate the imperative need to include coastal MPAs in the MCCETP network initiative, since connectivity and resilience of coral reefs in the equatorial eastern Pacific region rely heavily on coastal MPAs.

## Introduction

Coral reefs in the eastern Pacific span from the Gulf of California to Easter Island (Rapa Nui) and from the oceanic islands off western Mexico, Costa Rica, Panama, Colombia and Ecuador to the west coast of America [1]. In this region, coral reefs thrive under extreme environmental conditions, from short-term variability in sea temperature to long-term temperature excursions accompanying El Niño/La Niña episodes [1, 2]. In addition, there are nutrient pulses during seasonal upwelling, strong turbulent vertical mixing and entrainment of subsurface water into the surface layer driven by mountain gap-winds [3]. Low and variable pH values have also been reported in recent studies [4–8]. The synergy between harsh environmental conditions and low accommodation space has restricted the production of skeletal calcium carbonate, resulting in small, patchy reefs prone to erosion [9].

After mild to severe anthropogenic and natural disturbances, the recovery of coral reefs in the eastern Pacific has proceeded at a variable pace following several paths. Reef recovery is closely related to the type and extent of disturbance, reef characteristics, connectivity, and anthropogenic influences via the implementation of management strategies, law enforcement, and the establishment of marine protected areas (MPAs) [10–17].

The Marine Conservation Corridor of the Eastern Tropical Pacific (MCCETP), formally established in 2004, is an instrument for the conservation and sustainable use of the biological diversity in the equatorial eastern Pacific region. MPAs offshore from Costa Rica (Cocos Island), Panama (Coiba Island), Colombia (Malpelo Island and Gorgona Island) and Ecuador (Galapagos Islands) support robust coral communities and were chosen as critical nodes in marine dispersal pathways [12]. The MCCETP represents an international effort to promote long-term conservation and management within the equatorial eastern Pacific region. However, it is still unknown whether the MPAs (i.e. Cocos, Coiba, Malpelo, Gorgona, and Galapagos islands) included in the MCCETP form a well-connected network, which is expected to be functionally more effective than isolated MPAs [18] to guarantee the long-term conservation of marine biodiversity.

Connectivity, defined as the dispersal of individuals from one subpopulation to another [19], is relevant as it protects biodiversity, increases resilience of coral reef ecosystems and enhances their recovery after disturbances [10–17]. Different methods are currently being used to assess the connectivity of corals, from addressing gene flow among populations [20–22] to biophysical modeling [20, 23–27]. While empirical methods are relatively expensive and require intensive sampling, biophysical modeling offers the possibility to study the connectivity of corals by means of tracking numerous virtual larvae over a wide range of spatio-temporal scales and under several scenarios. This work assesses the potential connectivity among 30 MPAs (including the five MPAs of the MCCETP) harboring coral reefs within the equatorial eastern Pacific region using a biophysical model (i.e. a Lagrangian particle-tracking algorithm coupled with an ocean-circulation numerical model). This study aims to: a) identify the extent of connectivity among MPAs across the equatorial eastern Pacific region; b) evaluate the major spatial connectivity patterns; c) determine the importance of each site and pathway for local and regional connectivity; and d) suggest areas that might be prioritized for marine conservation efforts in the region.

## Materials and methods

### Hydrodynamic model

The Regional Ocean Modeling System (ROMS_AGRIF v3.1.1) [28] was used to simulate the ocean circulation in the equatorial eastern Pacific region. It is a free-surface, primitive equations model, discretized in terrain-following vertical and orthogonal curvilinear horizontal coordinates using high-order numerical algorithms [29]. The computational domain covers the region within 4°S-18°N and 76–114°W with resolution of 1/12° (~9 km) in the horizontal and 32 sigma layers in the vertical. The model grid, atmospheric forcing, initial and open lateral boundary conditions were built using the ROMSTOOLS package [30]. The model bathymetry was based on ETOPO1 [31]. Monthly-mean atmospheric forcing was gathered from COADS09 climatology [32] and QuikSCAT 2000–2007 [33]. Initial and open lateral boundary conditions were based on WOA09 climatology [34]. At the open lateral boundaries, an active, implicit, upstream-biased radiation condition was used [35]. In the case of inflow, the solution at the boundary was nudged toward the climatology. The model was run (with internal and external time-steps of 300 and 10 s, respectively) for ten years to obtain a nearly repeating annual cycle (climatological simulation). The spin-up phase was about three years.

### Larval dispersal simulation

**Fig 1:**
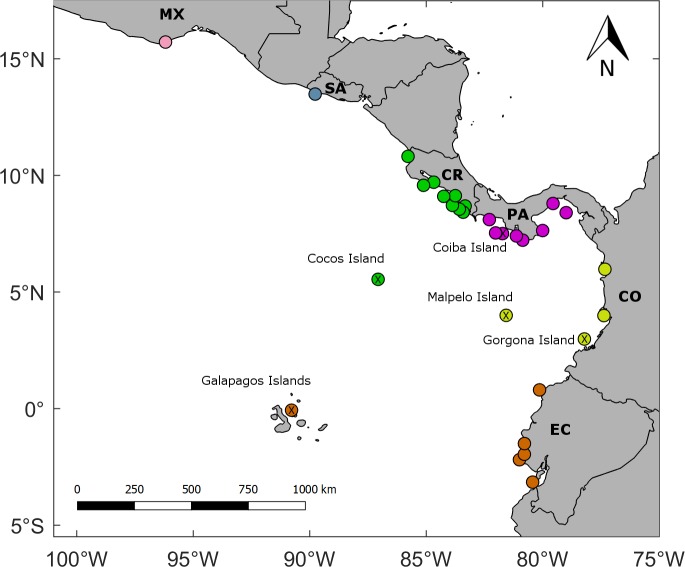
Marine protected areas (MPAs) with coral reef in the equatorial eastern Pacific region. MPAs color-coded by country: Pink (Mexico (MX): Huatulco Bays); Gray (El Salvador (SA): Los Cobanos); Green (Costa Rica (CR): Santa Rosa, Cabo Blanco, Playa Blanca, Manuel Antonio, Marino Ballena, Caño Island, Corcovado, Río Oro, Piedra Blanca, and Cocos Island); Purple (Panama (PA): Golfo de Chiriquí, Isla Montuosa and Banco Hannibal, Coiba Island, Veraguas, Cerro Hoya, Iguana Island, Taboga-Uraba, and Las Perlas); Yellow (Colombia (CO): Utria, Malaga, Gorgona Island, and Malpelo Island); and Orange (Ecuador (EC): Galapagos Islands, Galera-San Francisco, Machalilla, El Pelado, Santa Elena, and Santa Clara). The MCCETP MPAs are marked with a cross inside the circle. For details about the MPAs, refer to [17]. All maps were made by using the GSHHS coastline database [51].

Passive larval dispersal was simulated using the Lagrangian algorithm Ichthyop v3.2 [36]. Advection was calculated using a Runge-Kutta fourth-order numerical scheme. Horizontal diffusion was added by means of a random walk for individual larvae to account for sub-grid scale hydrodynamics not resolved by the ocean-circulation model [37, 38]. Current MPAs (30 polygons) with coral reefs [17] were used as release and recruitment zones (Fig 1). In each polygon, particles were randomly released between the surface and 30-m depth, and the position of each particle was recorded every day. Because of the relatively large coral reef spawning period in the equatorial eastern Pacific region [1], particles were released the first day of every month for one year using the three-dimensional velocity fields (daily-averages) of the last year from the climatological simulation performed with the hydrodynamic model. There were twelve release events per MPA, each including 10000 particles, which yields 120000 particles per MPA per year. We assumed that the potential contribution of each MPA to regional connectivity is independent of MPA size and reef extension.

Particles were tracked assuming pelagic larval duration (PLD) values of 40 and 130 days. PLD included a pre-competency period of 10 days (during which larvae cannot be recruited) and competency periods of 30 and 120 days, respectively. These periods correspond to the maximum larval competency reported for different coral species [39], and allow research on how different larval competency periods affect connectivity. When particles were located in a MPA polygon between the pre-competency and the end of competency period (i.e. 40 and 130 days), they were considered as recruited. After the PLD, larvae were considered dead. As a first assessment, we considered neither larval vertical migration (only passive dispersal), nor mortality.

### Connectivity metrics and graph theory

Connectivity matrix *Cij* was calculated as the number of larvae originating from a release MPA *j* that ended up in recruitment MPA *i* [38, 40]. Self-recruitment was defined as the percentage of larvae retained or recruited back to the release MPA (the main diagonal of the matrix). Subsidiary recruitment, on the other hand, was defined as the percentage of larvae recruited in a MPA other than the release MPA (outside the main diagonal). We also used a graph theory analysis that consists of a set of nodes (i.e. MPAs) and linkages (larval exchange) between all connected nodes. Betweenness centrality and neighborhood analysis were used to elucidate key ecological processes [38, 41]. Following Andrello et al. [38] and Treml et al. [41], betweenness centrality is the proportion of shortest paths between all MPA pairs that pass through a particular MPA; this metric highlights the ‘most commonly used’ route, representing major pathways, and helps to identify key stepping-stone MPAs. Node neighborhood, on the other hand, refers to the set of MPAs connected immediately upstream (In-degree of nodes) and downstream (Out-degree of nodes). Comparing the connection between individual MPAs provides insight on the degree to which the MPA acts as source or sink of larvae. Lastly, the Louvain method [42] was used to detect communities (i.e. a set of MPAs) within a same large graph. This analysis is important as it helps to highlight unknown functional MPA sets that may have seminal relevance to regional connectivity.

## Results

### Connectivity matrix

**Fig 2:**
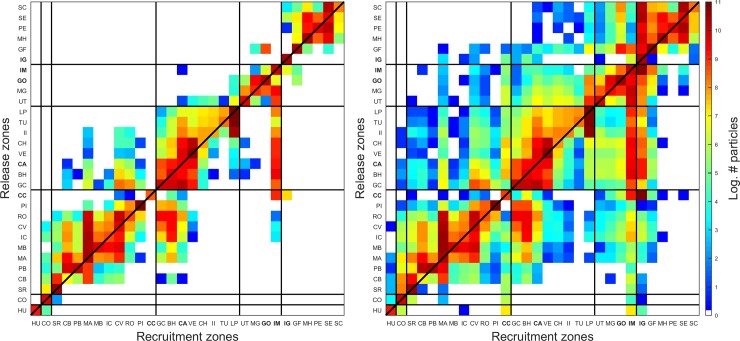
**Connectivity matrices for PLD of 40 days (left panel) and 130 days (right panel).** Solid lines separate MPAs of Mexico, El Salvador, Costa Rica, Panama, Colombia and Ecuador. Mexico: HU (Huatulco Bays); El Salvador: CO (Los Cobanos); Costa Rica: SR (Santa Rosa), CB (Cabo Blanco), PB (Playa Blanca), MA (Manuel Antonio), MB (Marino Ballena), IC (Caño Island), CV (Corcovado), RO (Rio Oro), PI (Piedra Blanca), CC (**Cocos Island**); Panama: GC (Golfo de Chiriquí), BH (Isla Montuosa and Banco Hannibal), CA (**Coiba Island**), VE (Veraguas), CH (Cerro Hoya), II (Iguana Island), TU (Taboga-Uraba), LP (Las Perlas); Colombia: UT (Utria), MG (Malaga), GO (**Gorgona Island**), IM (**Malpelo Island**); and Ecuador: IG (**Galapagos Islands**), GF (Galera-San Francisco), MH (Machalilla), PE (El Pelado), SE (Santa Elena), SC (Santa Clara). Names in bold indicate MPAs of the Marine Conservation Corridor of the Eastern Tropical Pacific (MCCETP).

In the two PLD scenarios (40 and 130 days) the number of particles was high across or close to the diagonal of the connectivity matrix, indicating that high self- and subsidiary recruitment between relatively close MPAs were the predominant processes (Fig 2). Both PLD scenarios also revealed that the transport of larvae tends to be southward. Additionally, the recruitment intensity and dispersal distances increased with PLD such that, in contrast to the high connectivity among MPAs from southern Costa Rica and northern Panama in the short PLD scenario, MPAs from the southern Mexico, El Salvador, Costa Rica, Panama, Colombia and Ecuador became relatively well connected in the long PLD scenario.

### Genetic and demographic connectivity

**Fig 3:**
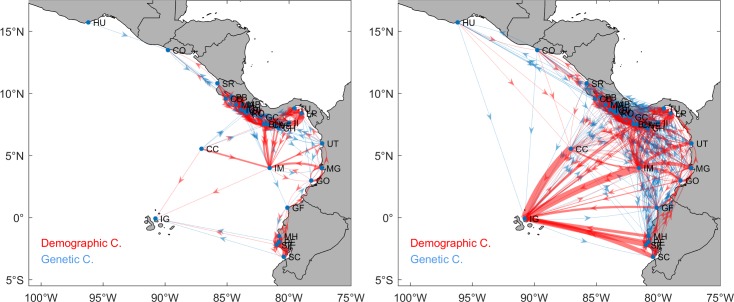
**Genetic and demographic connectivity for PLD of 40 days (left panel) and 130 days (right panel).** Line width represents larval transport intensity. Blue and red lines correspond to potential genetic (*p* ≤ 0.001) and demographic (*p* > 0.001) connectivity, respectively. All maps were made by using the GSHHS coastline database [51].

The connectivity matrices revealed that the larval exchange among MPAs may potentially involve from one to several thousand larvae per year; therefore, its potential effect on population dynamics likely varies markedly. Probability threshold of 0.001 [43] was used to differentiate between genetic and demographic connectivity. While the former affects allele frequency only, the latter also affects population growth rate or specific vital rates (such as survival and birth). According to our results, 64% and 51% of all connections were demographically relevant for PLD values of 40 and 130 days, respectively (Fig 3).

### Larval dispersal pathways

**Fig 4:**
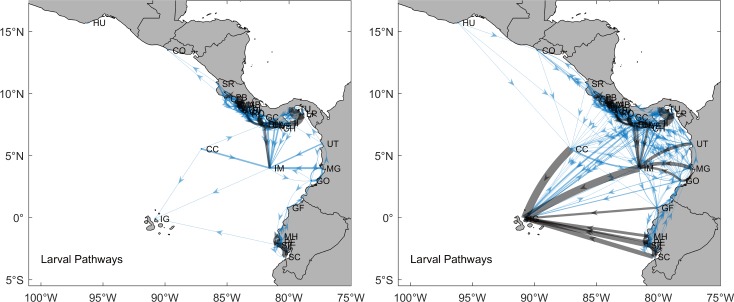
**Larval transport pathways for PLD of 40 days (left panel) and 130 days (right panel).** Line width denotes larval transport intensity. Black lines represent the major larval transport pathways (≥ 10% of larvae produced in the MPA). Blue lines indicate secondary larval transport pathways (< 10% of larvae produced in the MPA). All maps were made by using the GSHHS coastline database [51].

Highlighting of the highest larval fluxes among MPAs (≥ 10% of larvae produced in a given MPA) (Fig 4) showed that in the short PLD scenario, major larval transport pathways were located along the coast of Costa Rica (except Santa Rosa), Panama (all MPAs) and southern Ecuador (Machalilla, El Pelado, Santa Elena, and Santa Clara). In this PLD scenario, a consistent larval flux was also located from Isla Montuosa and Banco Hannibal (Panama) to Malpelo Island (Colombia). In the long PLD scenario, the number of new potential pathways increased, especially between the coastal zone and oceanic islands, as well as among them. Particularly, coastal MPAs in Panama, Colombia, and Ecuador supplied larvae to Malpelo Island and Galapagos Islands, while Cocos and Malpelo islands supplied larvae to the Galapagos Islands. Regarding the MCCETP, four of the five MPAs (i.e. Cocos Island, Coiba Island, Gorgona Island, and Galapagos Islands) were part of major larval transport pathways in the long PLD only. In this case, the unidirectional transport of larvae created a downstream larval flow pattern starting at Coiba (Panama) and Gorgona (Colombia) islands, and ending in the Galapagos Islands (Ecuador).

### Strong components

**Fig 5:**
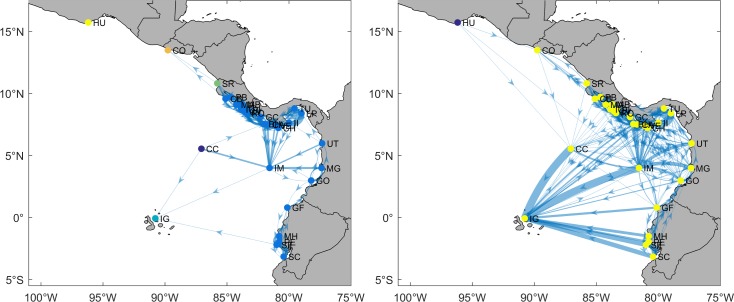
**Strong components for PLD of 40 days (left panel) and 130 days (right panel).** Line width stands for larval transport intensity, with the wider line indicating more intense larval transport. Colors represent the different strong components. All maps were made by using the GSHHS coastline database [51].

The graph component analysis showed relatively high connectivity in both PLD scenarios (Fig 5). Six and two strong components were recognized for the PLD of 40 and 130 days, respectively. Five single-MPA components (Huatulco Bays, Los Cobanos, Santa Rosa, Cocos Island, and Galapagos Islands) and one multi-MPA component (the remaining 25 MPAs) were detected for the short PLD. In contrast, one single-MPA component (Huatulco Bays) and one multi-MPA component (the other 29 MPAs) were detected for the long PLD. It is worth noting that Huatulco Bays (Mexico) was demographically isolated in the short PLD and partially connected (upstream MPA) in the long PLD, which could lead to important consequences regarding its persistence. On the other hand, the structure of MCCETP MPA components changed in the long PLD scenario, becoming a fully connected network. Nevertheless, the larval flow was mainly unidirectional.

### Betweenness centrality

**Fig 6:**
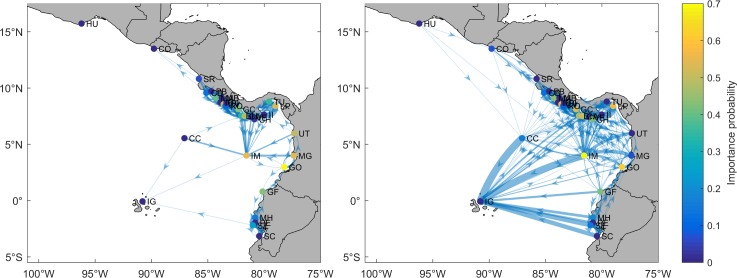
**Betweenness centrality for PLD of 40 days (left panel) and 130 days (right panel).** Line width stands for larval transport intensity, with the wider line indicating more intense larval transport. All maps were made by using the GSHHS coastline database [51].

High betweenness centrality revealed MPAs that were frequently used as critical nodes in the graph, and that may play a key ecological role as stepping-stone localities. In general, differences in PLD had marginal impact on betweenness centrality (Fig 6). In the short PLD scenario, betweenness centrality was higher in Corcovado (Costa Rica), Las Perlas (Panama), Malaga, Malpelo Island and Gorgona Island (Colombia), where the centrality values ranged from 0.51 to 0.79. In the long PLD scenario, the overall structure of betweenness centrality remained virtually unchanged, but for two major changes: there was a slight increase in intensity (except for Malaga and Gorgona islands, where it decreased), whereas Golfo de Chiriquí and Coiba Island increased their relevance in the structure. It is worth noting that two MCCETP MPAs (i.e. Gorgona and Malpelo islands) appeared as permanent stepping-stones in the equatorial eastern Pacific region.

### Neighborhood and resilience

**Fig 7:**
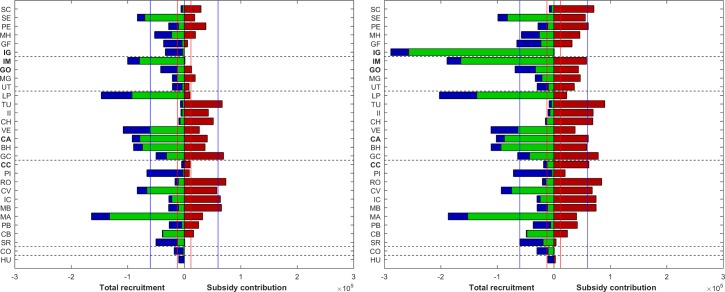
**Number of larvae exchanged for PLD of 40 days (left panel) and 130 days (right panel).** Green bars = subsidiary recruitment (or in-degree); Blue bars = self-recruitment; Red bars = subsidiary contribution (or out-degree). Dashed lines separate MPAs of Mexico, El Salvador, Costa Rica, Panama, Colombia and Ecuador. Red and blue lines represent 10% and 50% of larval production from each MPA, respectively.

Neighborhood analysis provided insight about the ecological role (i.e. sources and sinks) and level of resilience of each MPA (Fig 7, S1 and S2 Figs). For the short PLD, three source MPAs were detected in Costa Rica (Marino Ballena, Caño Island, and Rio Oro) and two in Panama (Golfo de Chiriquí and Taboga-Uraba). In addition, two sink MPAs were detected in Costa Rica (Manuel Antonio and Corcovado), four in Panama (Isla Montuosa and Banco Hannibal, Coiba Island, Veraguas, and Las Perlas), one in Colombia (Malpelo Island), and one in southern Ecuador (Santa Elena). In the long PLD scenario, on the other hand, the number of source and sink MPAs increased. Already recognized source and sink MPAs remained strongly active, but increased their subsidiary recruitment and contribution level. In addition, new source MPAs were detected in Costa Rica (Corcovado and Coco Island), Panama (Coiba Island, Cerro Hoya, and Iguana Island), and Ecuador (El Pelado and Santa Clara). A new sink MPA was also detected in Ecuador (Galapagos Islands). It should be noted that the Galapagos Islands are one of the largest sinks of larvae in the equatorial eastern Pacific region.

In general, high/low subsidiary- and self-recruitment indicate high/low resilience potential (Fig 7). When both subsidiary- and self-recruitment are taken into account, all sink MPAs detected in the short PLD were considered as resilient. This is also true for Piedra Blanca (Costa Rica) because of its very high self-recruitment. In the long PLD scenario, already resilient MPAs displayed a pronounced increase in their resilience level, while new, potentially resilient MPAs emerged in Costa Rica (Santa Rosa), Panama (Golfo de Chiriquí), Colombia (Gorgona Island), and Ecuador (Galapagos Islands and Galera-San Francisco). In general, increasing PLD enhanced the potential resilience of MPAs particularly in the MCCETP (except Coco Island, which was one of the least resilient MPAs across the equatorial eastern Pacific region). On the other hand, low resilience MPAs were also located in Mexico (Huatulco Bays), Panama (Iguana Island and Taboga-Uraba), and Ecuador (Santa Clara).

### Communities

**Fig 8:**
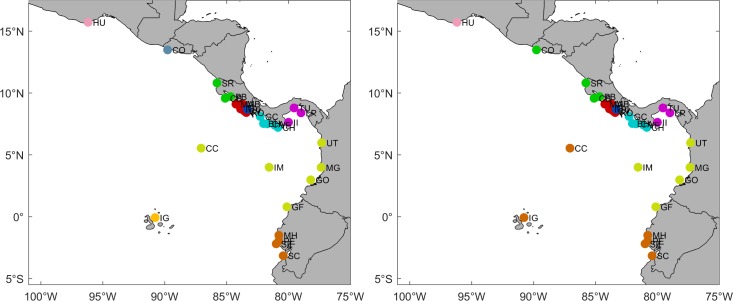
**Communities according to the Louvain method for PLD = 40 days (left panel) and PLD = 130 days (right panel).** Colors represent different communities detected by the algorithm. All maps were made by using the GSHHS coastline database [51].

According to the Louvain method, in the two PLD scenarios, the maximum modularity was reached in two passes (Fig 8). In the short PLD scenario, the pass of maximum modularity recognized ten communities. Mexico (Huatulco Bays), El Salvador (Los Cobanos), Costa Rica (Piedra Blanca), and Ecuador (Galapagos Islands) formed isolated communities. In addition, MPAs located along the coast of Costa Rica were divided in two communities: northern Costa Rica (Santa Rosa, Cabo Blanco, and Playa Blanca) and southern Costa Rica (Manuel Antonio, Marino Ballena, Caño Island, Corcovado, and Rio Oro). Panama was also divided in two communities: northern Panama (Golfo de Chiriquí, Isla Montuosa and Banco Hannibal, Coiba Island, Veraguas, and Cerro Hoya) and southern Panama (Isla Iguana, Taboga-Uraba, and Las Perlas). Colombian MPAs, along with Costa Rica and Ecuador (Utria, Malaga, Gorgona Island, Malpelo Island, Cocos Islands, and Galera-San Francisco) formed a larger community. Finally, Ecuador (Machalilla, El Pelado, Santa Elena, and Santa Clara) formed another community. In the long PLD scenario, eight communities were detected. The main differences between short and long PLD communities reside in the inclusion of El Salvador (Los Cobanos) in the northern Costa Rica community and the expansion, with the inclusion of Cocos Island and Galapagos Islands, of the coastal community located in Ecuador. Regarding the MCCETP MPAs, the two PLD scenarios excluded Coiba Island as an independent community, while the rest of the MCCETP MPAs formed larger communities. The short PLD highlighted the community structure among Cocos, Malpelo and Gorgona islands, while the long PLD scenario revealed the community structure between Cocos Island and Galapagos Islands, as well as between Malpelo and Gorgona islands.

## Discussion

Our results suggest that the MPAs located in the equatorial eastern Pacific region formed a relatively well-connected network. At least 40% of the annual larval production in MPAs was able to reach other MPAs (~24%) or remained within the boundaries of the source MPA (~16%). Subsidiary- and self-recruitment clearly increase (~40 and ~19%, respectively) with PLD, which is a key interspecific parameter for assessing connectivity [44]. In the present work, the 40 and 130-day PLD scenarios may encompass maximum potential connectivity of the main coral reef framework building species (*Porites*, *Pavona*, and *Pocillopora*; refer to [1] for a detailed account) in the study area. A long PLD tends to increase recruitment because larvae have more time to reach distant MPAs, but larvae may also have more time to be recruited back to the MPA of origin, provided that oceanographic conditions are adequate. Contrary to early ideas, recent connectivity studies [24, 26] have suggested that self-recruitment and nearby larval transport prevail across coral species, even for broadcast spawner taxa (reducing the connectivity among relatively distant areas). Our results also suggest that high potential larval transport occurs among relatively close (<100 km apart) MPAs, especially along the coast of Costa Rica and Panama. Nevertheless, the probability of larval transport across relatively distant MPAs (>500 km apart) increases when PLD is high and when larval release is synchronized with particular oceanographic current patterns. Considering the potential connectivity scenario observed among close MPAs in the equatorial eastern Pacific, an MPA network should be carefully designed and managed to ensure the persistence of coral reefs at a regional scale, particularly between southern Mexico and Costa Rica, as well as between southern Panama and Ecuador.

Overall, the main larval transport pathways coincide with the mean direction and strength of ocean surface currents (S3 Fig). Indeed, larval fluxes tended to mirror the direction of currents, creating permanent routes between certain MPAs. The main larval routes were located along the coast of Costa Rica, Panama and southern Ecuador, following the deviations of the North Equatorial Counter Current (NECC). When the NECC impacts the continent (between Costa Rica and Panama), one branch deviates to the north, forming the Costa Rica Coastal Current (CRCC) and the another branch flows southward forming the Panama Current (PC), later joining the South Equatorial Current (SEC). As such, larval transport in Costa Rica and Panama mainly occurs northward and southward, respectively. In contrast, the arrival of the CRCC to Huatulco Bays (Mexico) and its subsequent incorporation into the Tehuantepec Bowl and the North Equatorial Current is restricted mostly to the winter season, which explains the relative isolation of this MPA. Southern Ecuador is located in a transition zone, where the Humboldt Current and the PC reach the SEC [45]. Consequently, the Santa Elena MPA (Ecuador) represents a convergence spot for larval transport. When PLD increases, new pathways emerge among MPAs located along the coast and near oceanic islands (i.e. Cocos Island (Costa Rica), Malpelo Island (Colombia), and Galapagos Islands (Ecuador), largely mediated by the southern deviation of the NECC. Even if these main larval transport pathways move massive quantities of larvae (> 12000 larvae/year), the unidirectional larval flux (weakly connected network) produces strict downstream larval transport patterns.

The genetic and demographic connectivity were analyzed separately because of their differences for management and conservation purposes. Genetic connectivity is generally useful for biodiversity conservation and biogeographic purposes, whereas demographic connectivity allows for defining whether populations are potentially sustainable and, consequently, should be taken into account in the design of MPA networks [13]. The connectivity threshold (0.001) used by Treml and Halpin [43] determines how larval transport may affect coral reef populations; a low migration rate (≤0.001) between populations is generally sufficient to maintain a relative genetic similarity between them, but not for creating demographic connections. However, since the demographic parameters of local populations in each MPA remain unknown, this assumption should be taken with caution.

The graphic-theoretical analysis of all connections revealed that the MPA network was not fully connected under the short PLD scenario, a finding that involves large-scale consequences for population genetics and adaptation. For instance, Huatulco Bays (Mexico) and Cocos Island (Costa Rica) are considered as strict gene sources (no subsidiary recruitment) for the rest of the equatorial eastern Pacific region; therefore, these may not receive any new adaptive alleles from other MPAs. However, the long PLD scenario reveals a fully connected network, which is consistent with genetic studies [21, 22], suggesting that gene flow is high across the region, particularly along the coast and between the coast and oceanic islands (i.e. Cocos Island, Malpelo Island, and Galapagos Islands). In contrast, demographic connectivity is relevant to maintain population dynamics at a large scale. Even when both PLD scenarios showed a demographic connectivity above 50%, MPAs appeared as not fully connected. Under the short PLD scenario, for example, Huatulco Bays and Cocos Island were weakly connected (or isolated), whereas in the long PLD scenario, only Huatulco Bays remained as a strict source. Under both PLD scenarios, Huatulco Bays could potentially be connected to MPAs located in northern Mexico (i.e. Marias Islands or Revillagigedo Islands) or other coral reefs (i.e. Zihuatanejo) [25, 46] not included in the current study, which could enhance the genetic variability and/or demographic sustainability.

In addition to direct connections, connectivity among MPAs in the equatorial eastern Pacific may result from stepping-stone or multi-step connections. The stepping-stone approach represents the transport of larvae between two MPAs not directly connected, with a third MPA acting as a central node (in a multi-generational way). Our results indicate that several MPAs have high betweenness centrality, meaning they are mandatory pathways for larval dispersal. In both PLD scenarios, Corcovado (Costa Rica), Las Perlas (Panama), Gorgona Island and Malpelo Island (Colombia) act as key pathway nodes for preserving network connectivity across the equatorial eastern Pacific region. The overall network structure and oceanic currents jointly increase the betweenness centrality of these particular MPAs. For instance, a high number of MPAs located along the coast of Costa Rica and Panama use Malpelo Island as a pathway to reach MPAs along the coast of Ecuador or the Galapagos Islands, but neither Cocos Island nor the Galapagos Islands play a key role as connectivity nodes in the equatorial eastern Pacific region. According to the betweenness centrality, Malpelo and Gorgona islands, included in the MCCETP initiative, can be considered as the most “important” MPAs in the network, since most of the connectivity among MPAs of the region passes through them. Moreover, it is reasonable to state that MPAs in northwestern Mexico may be linked to MPAs in the equatorial eastern Pacific region via Huatulco Bays, thus turning the latter into an MPA with one of the highest betweenness centrality potential in the entire eastern Pacific region.

The heterogeneity in the structure of larval sinks and sources (number and location) is a key factor for metapopulation dynamics and resilience. Our results show that the structure of larval sink MPAs remains relatively constant under both PLD scenarios, except for the Galapagos Islands, which sharply increase its subsidiary recruitment under the long PLD scenario and become the major sink MPA of the equatorial eastern Pacific region. However, the high level of imported larvae is likely a consequence of the large extension of the Galapagos Islands MPA (~133000 km^2^) relative to the other MPAs (the second largest MPA is Malpelo Island with ~10000 km^2^). It is important to bear in mind that because of bathymetric restrictions, since hermatypic reef-building corals in the area rarely development below 50 m depth, just a portion of MPA habitats are suitable for coral recruitment and development, especially in the case of oceanic MPAs such as Malpelo Island and the Galapagos Islands [47]. In contrast, the structure of source MPAs varied according to the PLD scenario. Source MPAs were located mainly along the coast of Costa Rica and Panama under the short PLD scenario, but reached as far as southern Ecuador under the long PLD. Increasing the number of source MPAs is essential for coral reef resilience. Certainly, source MPAs are generally more important than sink MPAs and should be prioritized for protection [48], as they can supply larvae to MPAs with low self-recruitment levels or to those that have undergone anthropogenic or natural damage [13]. Recovery accounts of coral reef areas that were severely damaged during the 1982–83 and 1997–98 El Niño events in the equatorial eastern Pacific [1, 10–11, 16–17] may support the results obtained in this study.

Since population demographic parameters are unknown for most MPAs, quantifying subsidiary- and self-recruitment provides insight about the potential resilience of each MPA. Subsidiary- and self-recruitment are equally important when attempting to determine population sustainability, although in rather different ways. High self-recruitment (equivalent to the birth rate) implies that the death rate can be offset by the production of new individuals from the same population, while low self-recruitment indicates that the death rate cannot be fully compensated for and may lead to population decline (except if migration or asexual reproduction occurs). On the other hand, a high subsidiary recruitment can compensate for an eventual decline in the birth rate, or may foster recovery following disturbance. In addition, the lack of larval supply from other populations (low subsidiary recruitment) may lead to inbreeding and increases the risk of population extinction. Moreover, if a population is isolated (no subsidiary recruitment at all), adaptive alleles emerging from other populations, mainly by mutation, cannot reach it, which may reduce the potential for adaptation to the changing environmental conditions [37], such as those occurring in the equatorial eastern Pacific region. Therefore, high MPA connectivity and resilience may play a key role in the persistence and adaptability of coral reefs under the rapid climate change scenarios expected in the region [8]. Depending on PLD, larvae have more/less time to reach other MPAs (or to restock the releasing population), thus increasing/decreasing the resilience potential. The results reported here suggest that most MPAs are relatively resilient, especially under the long PLD scenario, but MPAs with low resilience such as Huatulco Bays, Cocos Island, Cerro Hoya, Iguana Island, Taboga-Uraba, and Santa Clara were also detected. Detailed studies on the coral reef populations of these MPAs should be conducted and, if our findings are corroborated, carefully designed management strategies should be implemented to preserve coral reef populations that are currently facing the synergistic threats of human activities and suboptimal environmental conditions experienced in the eastern Pacific region [7, 49].

Communities detected by the Louvain algorithm partially match the coral eco-regions (Mexico, Guatemala-El Salvador-Nicaragua, Costa Rica-Panama, Colombia-Ecuador, Cocos Island, Malpelo Island, and Galapagos Islands) described by Veron et al. [50]. However, the Costa Rica-Panama and Colombia-Ecuador eco-regions comprise at least five and two distinct communities, respectively. Moreover, Cocos and Malpelo islands are not considered as individual eco-regions, but rather as part of the Colombia-Ecuador eco-region. In addition, each of the MPA communities detected by the algorithm should receive separate consideration as a conservation zone, aimed at enhancing the maintenance and sustainable use of biological diversity in the equatorial eastern Pacific region. Except for a few cases involving Cocos Island (Costa Rica) and Galera-San Francisco (Ecuador), most of the MPA communities detected do not cross political boundaries, facilitating the potential of more effective management.

Selected as critical nodes in marine dispersal pathways, the MCCETP network represents an international effort to promote long-term conservation and management within the equatorial eastern Pacific region. However, according to our results, its relevance as a set of critical nodes is relatively marginal. For instance, only two of the five MPAs (i.e. Gorgona and Malpelo islands) in the MCCETP network stand as relevant stepping-stones for maintaining critical connections in the network. Likewise, a MPA already included in the MCCETP network rarely serves as a potential source of larvae for the region, even under the long PLD scenario (except for Cocos and Coiba islands). In addition, the resilience attained by MPAs in the MCCETP network varies according to the PLD. Under the short PLD the MCCETP network is not fully connected, producing a quasi-unidirectional larval flow from the coast to the oceanic islands, where the only resilient MPAs (Coiba and Malpelo islands) depend mostly on subsidiary recruitment from upstream MPAs (i.e. Costa Rica, Panama and Colombia). Under the long PLD, the MCCETP network becomes fully connected (even if the main larval flow remains virtually unchanged) and the resilience potential increases sharply (except for Cocos Island, which maintains a very low potential). However, the potential resilience is still based on subsidiary recruitment from MPAs located along the coast of Costa Rica, Panama, Colombia, and Ecuador, currently outside of the MCCETP network initiative. According to these results, it is imperative to include coastal MPAs in the MCCETP network initiative, not only because they include species-rich sites as suggested by recent inventories [12], but also because connectivity and resilience across the equatorial eastern Pacific region rely heavily on coastal MPAs for long-term conservation and sustainable use of biological diversity.

It is worth noting that the current evaluation of the MCCETP initiative is based solely on coral parameterization; hence, a multi-species connectivity approach is encouraged to assess the overall aim of the MCCETP. In addition, biological simplifications such as constant spawning periods, reef area, specific PLD, lack of mortality and overestimated recruitment areas, may have potentially biased the results [43, 44]. Additionally, the horizontal resolution of the hydrodynamic model (1/12°) does not resolve the small-scale turbulence affecting larval transport. Nevertheless, the results reported here represent a first step for assessing coral connectivity among MPAs in the equatorial eastern Pacific region and, therefore, should be considered as a workable hypothesis that deserves further testing using independent empirical information, including accurate biological and/or ecological field data.

## Supporting information

S1 Fig **Spatial representation of in-degree or subsidiary recruitment (left panel) and out-degree or subsidiary contribution (right panel) for PLD of 40 days.** Line width stands for larval transport intensity, with the wider line indicating more intense larval transport. All maps were made by using the GSHHS coastline database [51].
(TIFF)

S2 Fig **Spatial representation of in-degree or subsidiary recruitment (left panel) and out-degree or subsidiary contribution (right panel) for PLD of 130 days.** Line width stands for larval transport intensity, with the wider line indicating more intense larval transport. All maps were made by using the GSHHS coastline database [51].
(TIFF)

S3 Fig **Schematic representation of simulated surface ocean currents in winter (February, top left panel), spring (June, top right panel), summer (September, (bottom left panel), and autumn (November, bottom right panel).** CC (California Current), CRCC (Costa Rica Coastal Current), CRD (Costa Rica Dome), HC (Humboldt Current), NEC (North Equatorial Current), NECC (North Equatorial Counter Current), PC (Panama Current), PG (Panama Gyre), SEC (South Equatorial Current), TB (Tehuantepec Bowl). All maps were made by using the GSHHS coastline database [51].
(TIFF)
