# Supplementary figures and images for: Coral connectivity between equatorial eastern Pacific marine protected areas: A biophysical modeling approach
Source: PLoS One. 2018 Aug 29;13(8):e0202995. doi: 10.1371/journal.pone.0202995 (PMC6114865; doi:10.1371/journal.pone.0202995)

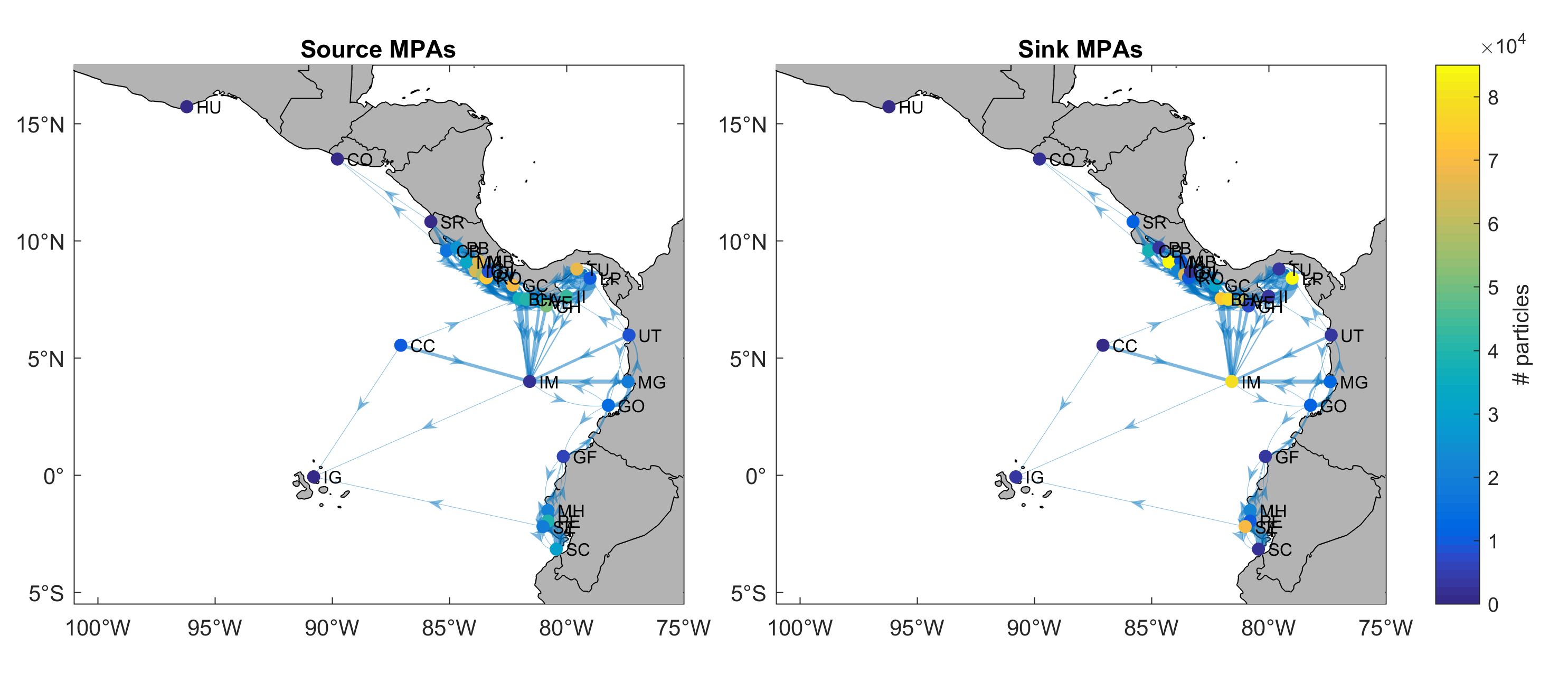

Supplement: S1 Fig — Spatial representation of in-degree or subsidiary recruitment (left panel) and out-degree or subsidiary contribution (right panel) for PLD of 40 days. Line width stands for larval transport intensity, with the wider line indicating more intense larval transport. All maps were made by using the GSHHS coastline database [51]. (TIFF) [file pone.0202995.s001.tiff]

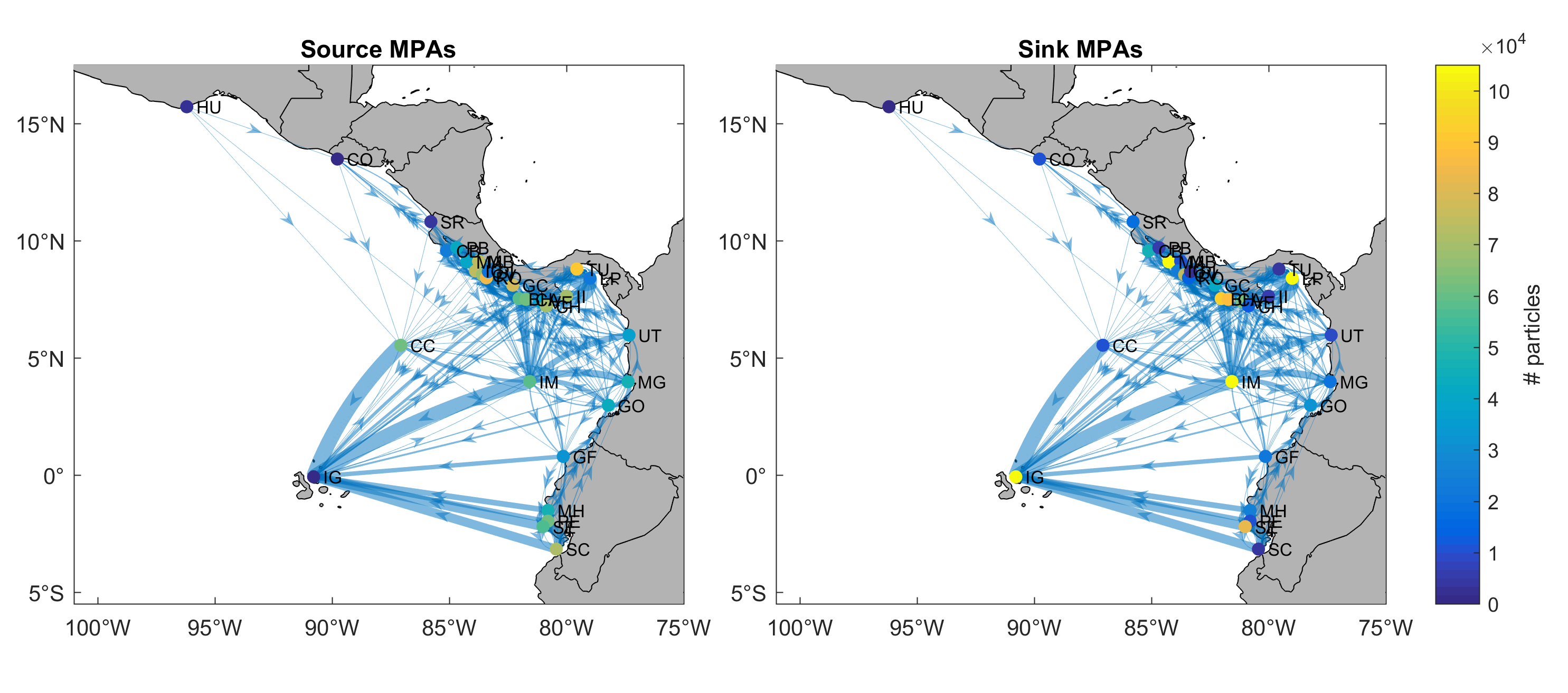

Supplement: S2 Fig — Spatial representation of in-degree or subsidiary recruitment (left panel) and out-degree or subsidiary contribution (right panel) for PLD of 130 days. Line width stands for larval transport intensity, with the wider line indicating more intense larval transport. All maps were made by using the GSHHS coastline database [51]. (TIFF) [file pone.0202995.s002.tiff]

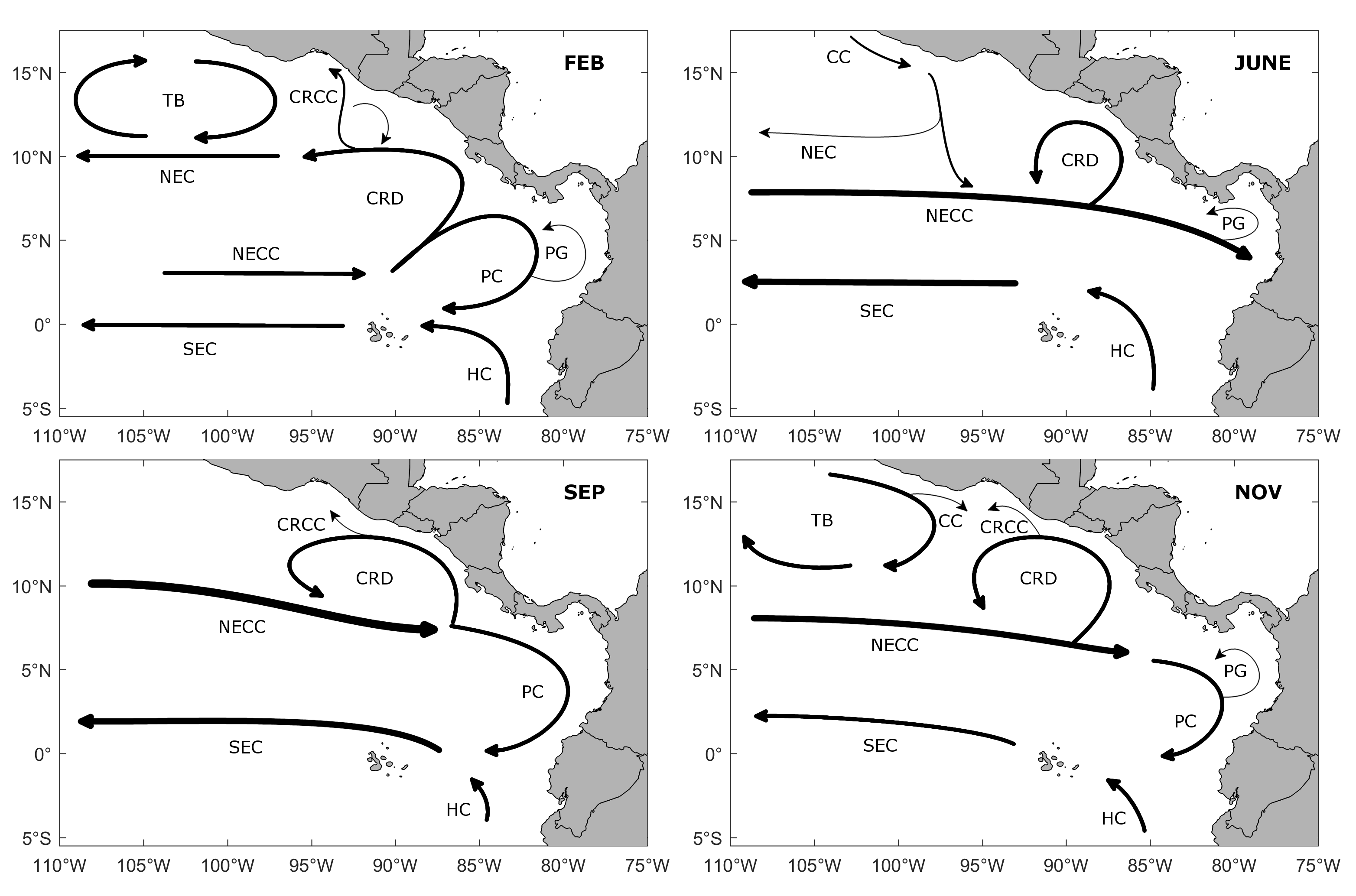

Supplement: S3 Fig — Schematic representation of simulated surface ocean currents in winter (February, top left panel), spring (June, top right panel), summer (September, (bottom left panel), and autumn (November, bottom right panel). CC (California Current), CRCC (Costa Rica Coastal Current), CRD (Costa Rica Dome), HC (Humboldt Current), NEC (North Equatorial Current), NECC (North Equatorial Counter Current), PC (Panama Current), PG (Panama Gyre), SEC (South Equatorial Current), TB (Tehuantepec Bowl). All maps were made by using the GSHHS coastline database [51]. (TIFF) [file pone.0202995.s003.tiff]
